# Diagnostik und Therapie bei Kindern und Jugendlichen mit chronischem Schmerz: Zeitliche Entwicklung potenziell gesundheitsgefährdender Maßnahmen

**DOI:** 10.1007/s00482-020-00506-5

**Published:** 2020-11-13

**Authors:** Felix Selent, Sabrina Schenk, Dunja Genent, Julia Wager, Boris Zernikow

**Affiliations:** 1 Deutsches Kinderschmerzzentrum, Vestische Kinder- und Jugendklinik Datteln, Universität Witten/Herdecke, Dr.-Friedrich-Steiner-Str. 5, 45711 Datteln, Deutschland; 2 Lehrstuhl für Kinderschmerztherapie und Pädiatrische Palliativmedizin, Fakultät für Gesundheit, Department für Humanmedizin, Universität Witten/Herdecke, Witten, Deutschland

**Keywords:** Risiko, Funktionelle Störung, Evidenzbasiert, Iatrogene Chronifikation, Bio-psycho-sozial, Risk, Functional disorder, Evidance based, Iatrogenic chronification, Bio-psycho-social

## Abstract

**Hintergrund und Ziel der Arbeit:**

Bei der Behandlung chronischer funktioneller Schmerzen im Kindes- und Jugendalter nimmt international sowohl die Anzahl an diagnostischen und therapeutischen Maßnahmen als auch ihre Invasivität zu. Studienziel ist die Erforschung der vor Beginn einer spezialisierten stationären Schmerztherapie durchgeführten, die pädiatrischen Patienten potenziell gefährdenden Maßnahmen in Deutschland.

**Material und Methoden:**

In einem retrospektiven Studiendesign wurden Patientenakten eines tertiären Kinderschmerzzentrums der Jahre 2004, 2008, 2012 und 2016 ausgewertet (*N* = 585). Neben diagnostischen und therapeutischen Maßnahmen wurden primäre Schmerzparameter und Patientencharakteristika erfasst. In einer interdisziplinären Expertenumfrage (*N* = 13) wurden die *Invasivität*, das *Risiko* und die *psychische Belastung* von Maßnahmen bewertet.

**Ergebnisse:**

Diagnostische und medikamentöse Maßnahmen nehmen bis 2012 zu. Ab 2012 lässt sich ein abnehmender Trend erkennen (*χ*^2^(3) = 11,708; *p* = 0,008). Die Invasivität (*χ*^2^(3) = 13,342; *p* = 0,004), das Risiko (*χ*^2^(3) = 13,135; *p* = 0,004) und die psychische Belastung (*χ*^2^(3) = 14,403; *p* = 0,002) durchgeführter Maßnahmen zeigen ein gleiches Veränderungsmuster. In der Gesamtstichprobe sind Patienten mit Bauch- oder Gliederschmerzen besonders gefährdet für hoch invasive und sehr risikoreiche Diagnostik.

**Diskussion:**

Eine Zunahme diagnostischer und therapeutischer Maßnahmen bei funktionellen Schmerzstörungen lässt sich nur bis 2012 beobachten. Bei bestimmten Patientengruppen kommen invasive, risikoreiche und die Psyche stärker belastende Maßnahmen häufiger zur Anwendung.

**Zusatzmaterial online:**

Die Online-Version dieses Beitrags (10.1007/s00482-020-00506-5) enthält vier weitere Tabellen. Beitrag und Zusatzmaterial stehen Ihnen auf www.springermedizin.de zur Verfügung. Bitte geben Sie dort den Beitragstitel in die Suche ein, das Zusatzmaterial finden Sie beim Beitrag unter „Ergänzende Inhalte“.

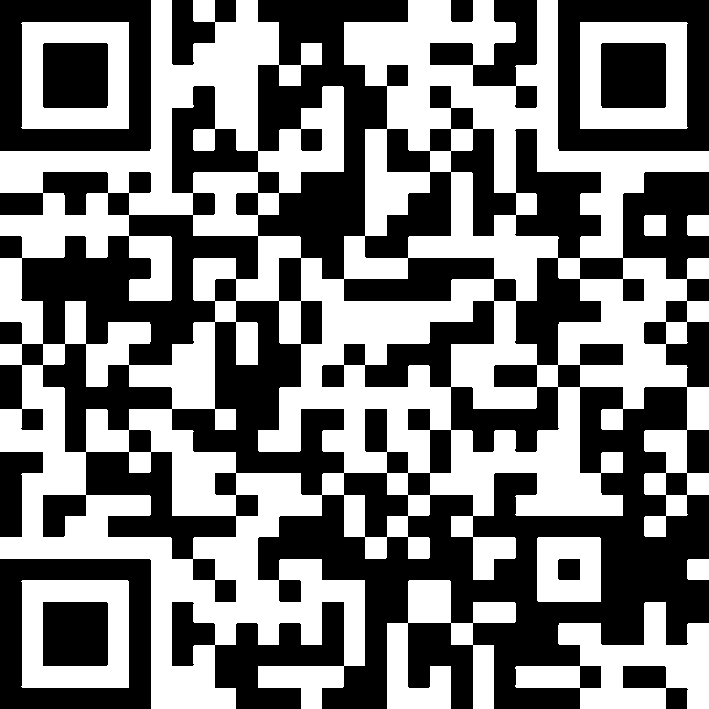

## Abstract (en)

**Background and objectives:**

Current research on the treatment of chronic pain in children suggests an increasing trend internationally in the quantity as well as invasiveness of diagnostic and therapeutic interventions. The objective of this research was to examine the interventions received by patients before starting specialized inpatient pain treatment.

**Materials and methods:**

A retrospective survey was conducted analyzing patient files from a tertiary children’s pain center from 2004, 2008, 2012 and 2016 (*N* = 585). In addition to diagnostic and therapeutic interventions, pain and patients’ characteristics were collected. The identified measures were subsequently evaluated by an interdisciplinary expert panel regarding their *invasiveness*, potential *risk* and degree of *mental burden*.

**Results:**

An increase in diagnostic measures and medication was found up to 2012. Thereafter, a decreasing trend was identified (*χ*^2^(3) = 11.708; *p* = 0.008). Invasiveness (*χ*^2^(3) = 13.342; *p* = 0.004), risk (*χ*^2^(3) = 13.135; *p* = 0.004) and mental burden (*χ*^2^(3) = 14.403; *p* = 0.002) showed the same pattern of change. Patients with abdominal and limb pain are particularly at risk for highly invasive and high risk diagnostics.

**Conclusions:**

Evidence for an increase in diagnostic and therapeutic measures in chronic pain was found up to 2012. Patients presenting with certain complaints receive comparably more invasive, risky and burdensome measures.

## Hintergrund

Chronische Schmerzen im Kindes- und Jugendalter stellen ein häufiges und zunehmendes Gesundheitsproblem dar. Zumeist können die Beschwerden auf eine funktionelle Störung zurückgeführt werden [1–4]. Dies setzt den Ausschluss einer aktiven körperlichen Grunderkrankung voraus. Es gibt evidenzbasierte Empfehlungen für das diagnostische Vorgehen bei rezidivierenden Schmerzen im Kindesalter [5–8]. Eine übersteigerte Diagnostik und Therapie bei anhaltenden Schmerzen kann zu einer iatrogenen Chronifizierung beitragen [9]. Sie ist für Patienten sowohl psychisch belastend als auch potenziell gefährlich [10]. Die aktuelle Studienlage belegt die Wirksamkeit multiprofessioneller multimodaler Ansätze, die auf invasive und potenziell gefährliche Maßnahmen weitgehend verzichten und stattdessen einen Schwerpunkt auf Patientenedukation, psychosoziale Maßnahmen und Steigerung der Selbstwirksamkeit legen [11–15].

Wissenschaftliche Untersuchungen aus den USA zeigen eine Zunahme der Anzahl diagnostischer und therapeutischer Maßnahmen sowie deren Invasivität beim Vorliegen einer funktionellen Schmerzproblematik im Kindes- und Jugendalter [16, 17]. Bislang wurde nicht überprüft, ob ein solcher Trend auch in Deutschland zu beobachten ist. In seltenen Fällen sorgt die Konstellation aus angewandter Diagnostik bzw. Therapie für ungünstigste Verläufe (Tab. 1).

**Table 1:** 

	Bauchschmerzen	Gelenkschmerzen
*Anamnese*	Weibliche Jugendliche (15 Jahre) mit seit 1Jahr bestehenden täglich mehrfach auftretenden Bauchschmerzen**;** mittlere Schmerzstärke „6“ und maximale Schmerzstärke „10“ (NRS 0–10)^a^	Männlicher Jugendlicher (17 Jahre) mit seit 4 Jahren bestehenden, dauerhaften Schmerzen wechselnder Gelenke**; **mittlere Schmerzstärke „4“ und maximale Schmerzstärke „8“ (NRS 0–10)^a^
	Schmerzbedingt kein Schulbesuch mehr möglich; starke Einschränkung im Alltag	10 % Schulfehltage; schmerzbedingt kein freies Gehen mehr möglich; Gebrauch von Unterarmgehstützen
*Diagnostik*	Psychologische Testuntersuchungen (Depressionsinventar für Kinder und Jugendliche und Angstfragebogen für Schüler) im Normbereich	Psychologische Testuntersuchungen zeigen leicht erhöhte Werte im Depressionsinventar für Kinder und Jugendliche
	2 × ÖGD^b^ ; 2 × Röntgenkontrastuntersuchung mittels Breischluckverfahren ohne pathologischen Befund	5 MRT (Sprunggelenk, Kiefer, Iliosakralgelenk, Fuß) sowie mehrfach umfangreiche Labordiagnostik zum Ausschluss einer rheumatologischen Grunderkrankung ohne pathologische Befunde
*Medikamentöse Therapie*	Gabe von Protonenpumpeninhibitoren, Metamizol und Domperidon ohne Beeinflussung der Schmerzen	12 verschiedene Medikamente (u. a. NSAR^c^, Opioide, Kortikosteroide, Methotrexat, 2 verschiedene monoklonale Antikörper) ohne Beeinflussung der Schmerzen
*Medizinische Interventionen*	Operation (Seit-zu-Seit-Duodenojejunostomie) und Anlage eines ZVK zur parenteralen Ernährung. Maßnahmen führen weder zur Schmerzreduktion noch zur Funktionsverbesserung	Lokalinfiltrationen des Iliosakralgelenks mit Lokalanästhetika und Steroiden erbrachten keine Schmerzreduktion
*Verlauf*	Stationäre multimodale Schmerztherapie zeigte guten Erfolg; Schulbesuch nach Entlassung ohne Schulfehltage	Während stationärer multimodaler Schmerztherapie Absetzen aller Medikamente, Steigerung der Belastbarkeit und der sportlichen Aktivitäten, Verzicht auf Unterarmgehstützen, keine Schmerzverstärkung bei Belastung
	Einschalten des Jugendamts wegen massiver häuslicher Problematik	Nachuntersuchung nach 12 Monaten: Aktivitäten des täglichen Lebens und Sport altersgerecht durchführbar
	Jetzt erfolgreiche Studentin	

^a^Numerische Rating-Skala (0 = kein Schmerz; 10 = maximaler Schmerz)

^b^Ösophagogastroduodenoskopie

^c^Nichtsteroidale Antirheumatika

Ziel dieser Studie ist die Untersuchung von Art und Anzahl eingesetzter Maßnahmen bei Kindern und Jugendlichen mit chronischen Schmerzen über einen Untersuchungszeitraum von 12 Jahren vor Beginn einer spezialisierten Schmerztherapie. Hierzu wird auch analysiert, ob das Risiko einer ausgedehnten Diagnostik bzw. Therapie für alle Kinder und Jugendlichen gleich groß ist oder ob sich Faktoren identifizieren lassen, die mit einem unverhältnismäßigen Einsatz von Maßnahmen assoziiert sind.

## Studiendesign und Untersuchungsmethoden

### Studiendesign

**Figure 1:**
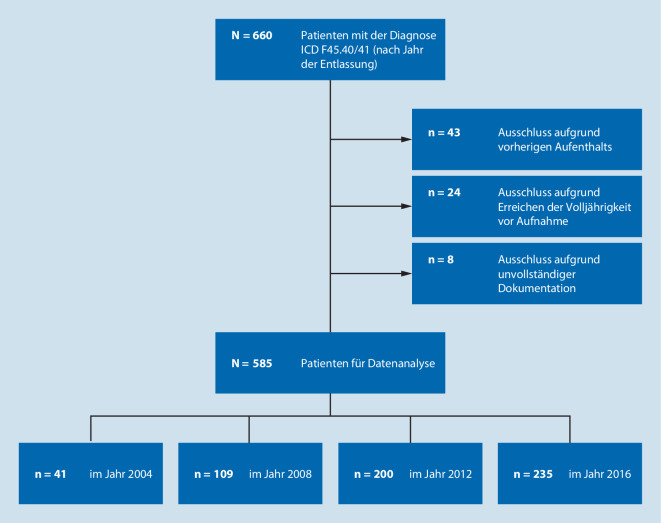

In die retrospektive Studie wurden Kinder und Jugendliche eingeschlossen, die aufgrund einer somatoformen oder chronischen Schmerzstörung (ICD-10: F45.40/F45.41) erstmalig in den Jahren 2004, 2008, 2012 oder 2016 zur stationären Schmerztherapie im Deutschen Kinderschmerzzentrum aufgenommen wurden. Von ursprünglich *n* = 660 möglichen Patienten wurden 75 aufgrund vorher definierter Kriterien ausgeschlossen (Abb. 1). Das Studienkollektiv umfasste somit *N* = 585 Kinder und Jugendliche mit chronischen funktionellen Schmerzen.

### Stichprobe

Die Patienten waren zum Zeitpunkt ihrer stationären Aufnahme im Durchschnitt 14,5 Jahre alt (*Spanne* = 3–17 Jahre; *SD* = 2,4) und überwiegend weiblich (*n* = 420, 72 %). Es wurden *n* = 251 (42,9 %) Patienten aufgrund einer anhaltenden somatoformen und *n* = 334 (57,1 %) Patienten aufgrund einer chronischen Schmerzstörung behandelt. Der Zeitraum von Beginn der Schmerzen bis zur Aufnahme auf die spezialisierte Schmerzstation ist über die betrachteten Jahre gleich geblieben (*MDN* = 28,4 Monate; *Spanne* = 1–184; *χ*^**2**^(3) = 7,053; *p* = 0,070).

### Daten der Aktenanalyse

Auf Basis der Patientenakten wurde neben primären Schmerzparametern (z. B. Schmerzdauer, -ort und -intensität sowie schmerzbezogene Beeinträchtigung) auch das Vorliegen somatischer sowie psychischer Begleiterkrankungen zum Zeitpunkt der stationären Aufnahme in eine Datenbank übernommen. Diese Daten werden seit 2003 standardisiert mithilfe des Deutschen Schmerzfragebogens für Kinder und Jugendliche erfasst, während des stationären Aufenthalts verifiziert und ergänzt [18].

Zudem wurde ermittelt, welche Art der **Diagnostik**, welche **Medikamente **und welche **schmerzspezifischen medizinischen Interventionen **(im Weiteren **„Bereiche“ **genannt) Patienten im Vorfeld der Aufnahme erhalten hatten. Im Datensatz wurden diese binär-nominal erfasst (Maßnahme erhalten: ja/nein).

Auf Grundlage der im klinischen Qualitätsmanagement genutzten Risikoprioritätszahlen zur Identifizierung und Bewertung steuerbarer Risikopotenziale [19, 20] wurden alle Maßnahmen bezüglich ihrer **„Invasivität“,** ihres **„Risikos“** und ihrer **„psychischen Belastung“** (im Weiteren **„Kriterien“** genannt) bewertet. „Invasivität“ wird hier definiert als Umfang der Integritätsverletzung des Körpers, „Risiko“ als die Wahrscheinlichkeit für das Auftreten und die Ausprägung unerwünschter Nebeneffekte und „psychischer Belastung“ als Grad der durch die Maßnahme bedingten Beeinträchtigung der Psyche. Ziel der Bewertung ist die Abschätzung eines **potenziellen** Schadens, den die Anwendung einer Maßnahme nach sich ziehen könnte. Diese Bewertung nahmen *n* = 13 Experten für jede einzelne Maßnahme getrennt in einer Online-Umfrage via SoSci Survey (SoSci Survey GmbH, München, Deutschland) vor dem Hintergrund einer schmerzbezogenen Indikation auf einer 5‑stufigen Likert-Skala vor (0 = keine bzw. 5 = sehr hohe Ausprägung eines der drei Kriterien; zzgl. der Option „keine Einschätzung möglich“). Die Experten sind medizinisch-therapeutisches Fachpersonal der Primär‑, Sekundär- bzw. Tertiärversorgung mit direktem Bezug zur Thematik wie auch medizinische Laien im Sinne Betroffener und Angehöriger (*n* = 8 ärztliche [Anästhesie/Schmerzmedizin, Kinderrheumatologie/-immunologie, Neuropädiatrie, Kinder‑/Jugendpsychiatrie, Kinderchirurgie/-orthopädie, Kinderradiologie] und *n* = 5 nichtärztliche Experten [Patienten/Angehörige, Pflegewissenschaften, Physiotherapie, MFA]). Als Ergebnis der Befragung lagen für jede Maßnahme aus den drei Bereichen (Diagnostik, Medikamente, Interventionen) Mittelwerte für die drei **Kriterien** „Invasivität“, „Risiko“ und „psychische Belastung“ vor (s. im elektronischen Zusatzmaterial, Tab. S1).

Patientenspezifisch wurden für alle durchgeführten Maßnahmen *Summenscores* der drei Kriterien **„Invasivität“, „Risiko“ **und** „psychische Belastung“ **für die Bereiche **„Diagnostik“, „Medikamente“** und **„medizinische Interventionen“** berechnet (*n* = 9 pro Patient). Abschließend wurden für jeden Patienten *Gesamtscores *für „**Invasivität“ **(der drei Bereiche), **„Risiko“ **(der drei Bereiche) und „**psychische Belastung“** (der drei Bereiche) gebildet (*n* = 3 Werte pro Patient; Tab. 2).

**Table 2:** 

Berechnung der Summen- und Gesamtscores anhand der Mittelwerte aus der Expertenumfrage nach folgendem Schema jeweils pro Patient						
		Summenscores^a^			Gesamtscores^b^	
		Bereiche				
		Diagnostik	Medikamente	Medizinische Interventionen	Rohwerte	>75 %^c^
*Kriterien*	Invasivität	DIA_INV_	MED_INV_	INTER_INV_	INV_GESAMT_	Hoch invasiv (>23,05)^d^
	Risiko	DIA_RISK_	MED_RISK_	INTER_RISK_	RISK_GESAMT_	Sehr risikoreich (>22,33)^e^
	Psychische Belastung	DIA_PSYCH_	MED_PSYCH_	INTER_PSYCH_	PSYCH_GESAMT_	Stark psychisch belastend (>26,35)^f^

^a^Aufaddierte Mittelwerte (MW) der Maßnahmen je Bereich und Kriterium

^b^Aufaddierte Summenscores je Kriterium

^c^Bezogen auf das Gesamtsample

^d^Bezogen auf Risikokinder (>75. Perzentil) *MW* = 30,72; *Spanne* = 23,05–63,98

^e^Bezogen auf Risikokinder (>75. Perzentil) *MW* = 29,70; *Spanne* = 22,33–55,10

^f^Bezogen auf Risikokinder (>75. Perzentil) *MW* = 34,36; *Spanne* = 26,35–71,62

### Statistische Analyse

Die statistischen Analysen wurden mithilfe des Statistikprogramms SPSS Version 25 (IBM, Armonk, USA) durchgeführt. Deskriptive Ergebnisse wurden unter Zuhilfenahme von Kruskal-Wallis- und Chi-Quadrat-Tests zuzüglich Post-hoc-Vergleichen auf statistische Zusammenhänge hin geprüft. Das Signifikanzniveau wurde auf *p* = 0,05 festgelegt. Bei multiplen Vergleichen wurden *p-*Werte nach Bonferroni-Holm korrigiert und angepasst. Effektstärken wurden nach Cohen berechnet [21].

Mögliche einflussnehmende Bedingungsfaktoren (Tab. 3) wurden mittels multivariater binär-logistischer Regressionsanalyse (Vorwärtsauswahl, bedingt) analysiert. Als abhängige Variable wurden diejenigen Summen- und Gesamtscores gewählt, die oberhalb des 75. Perzentils der Gesamtkohorte lagen. Scores oberhalb dieses Cut-offs wurden jeweils als „*hoch invasiv“*, „*sehr risikoreich“* oder „*stark psychisch belastend“* eingestuft, Patienten mit mindestens einem Score oberhalb des Cut-offs als *Risikopatienten* (Tab. 2).

**Table 3:** 

Bedingungsfaktoren	Beschreibung
Alter	In Jahren bei Schmerzbeginn
Geschlecht	Männlich (= 0) oder weiblich (= 1)
Versicherungsstatus	Gesetzlich (= 0) oder privat (= 1) krankenversichert
Intelligenz	IQ-Werte
Schmerzdauer	In Monaten vor stationärer Aufnahme
Hauptschmerzort	Kopf, Nacken, Thorax, Rücken, Bauch, Extremitäten
PPDI^a^	Beeinträchtigung im Alltag in den letzten vier Wochen vor stationärer Aufnahme
Schulfehltage (SFT)	Schulfehltage in den letzten vier Wochen vor stationärer Aufnahme
DIKJ^b^	T‑Wert ≥60 = klinisch auffällige Depressionswerte
AFS^c^	T‑Wert ≥60 in mindestens einem Subtest = klinisch auffällige Angstwerte

^a^Pediatric Pain Disability Index [22]

^b^Depressionsinventar für Kinder und Jugendliche [23]

^c^Angstfragebogen für Schüler [24]

### Ethik

Die Durchführung der Studie erfolgte unter vorliegendem positivem Votum der Ethik-Kommission der Universität Witten/Herdecke, Antrag Nr. 147/2018.

## Ergebnisse

### Entwicklung angewandter Maßnahmen über die Zeit

Die häufigsten **diagnostischen Untersuchungen** waren MRT (80 % der Patienten), EEG (44 %) und Sonographie (33 %). Patienten erhielten im Schnitt 1,77 MRT (Spanne: 1–11), 1,23 EEG (Spanne: 1–3) und 1,59 Sonographien (Spanne: 1–3). Die Anzahl diagnostischer Untersuchungen hat von 2004 bis 2012 signifikant zugenommen. Von 2012 auf 2016 zeigt sich ein signifikanter Abfall (*χ*^2^(3) = 11,708; *p* = 0,008).

97 % der Patienten erhielten vor Aufnahme **mindestens ein Medikament**. Am häufigsten wurden Ibuprofen (79 % der Patienten), Paracetamol (48 %) und Metamizol (42 %) eingenommen. Patienten erhielten im Schnitt 3,02 **verschiedene Medikamente** (Spanne: 1–10; *χ*^2^(3) = 5,350; *p* = 0,148). Der Medikamentenkonsum blieb über den Betrachtungszeitraum gleich. Hingegen veränderte sich das medikamentengruppenspezifische Einnahmeverhalten: Ibuprofen und Metamizol wurden häufiger eingenommen, Paracetamol seltener. Für Opioide ist seit 2008 ein abnehmender Trend zu beobachten (Tab. 4).

**Table 4:** 

Wirkstoff(-gruppe)^b^ *n*^c^ (%)^d^	2004 (*n* = 41)	2008 (*n* = 109)	2012 (*n* = 200)	2016 (*n* = 235)	Chi-Quadrat
Ibuprofen	28 (68,3)	72 (66,1)	154 (77,0)	209 (88,9)	*χ*^*2*^*(3)* *=* *28,448* *p* *<* 0,001*
Paracetamol	22 (53,7)	42 (56,9)	105 (52,5)	89 (37,9)	*χ*^*2*^*(3)* *=* *15,209* *p* *=* 0,010*
Metamizol	8 (19,5)	43 (39,4)	82 (41,2)	111 (47,2)	*χ*^*2*^*(3)* *=* *11,502* *p* *=* 0,027*
Benzodiazepine	3 (7,3)	5 (4,0)	2 (1,0)	2 (0,9)	*χ*^*2*^*(3)* *=* *11,932* *p* *=* 0,032*
Opioide	3 (7,3)	18 (16,5)	29 (14,5)	16 (6,8)	*χ*^*2*^*(3)* *=* *10,392* *p* *=* 0,032*
Acetylsalicylsäure	7 (17,1)	25 (22,9)	35 (17,5)	27 (11,5)	*χ*^*2*^*(3)* *=* *7,800* *p* *=* 0,050*

^*^Signifikant nach Bonferroni-Holm-Korrektur

^a^Einnahme vor Aufnahme

^b^Mehrfachverordnungen möglich

^c^*n* = Anzahl Patienten

^d^% = gültige Prozent

Von **schmerzspezifischen medizinischen Interventionen** waren 26 % der Patienten mit funktioneller Schmerzstörung betroffen. Die **Anzahl unterschiedlicher Interventionen** pro Patient ist insgesamt gering (im Mittel 1,19; Spanne: 1–3) und bleibt über den Untersuchungszeitraum unverändert (*χ*^2^(3) = 0,381; *p* = 0,944). Am häufigsten wurden Akupunktur (16 % der Patienten), Infiltrationsanästhesien (4 %) und Appendektomien (3 %) durchgeführt. Eine detaillierte Auflistung aller Maßnahmen und ihrer Häufigkeiten über die Jahre findet sich im elektronischen Zusatzmaterial in Tab. S2 und S3.

### Entwicklung der Invasivität, des Risikos und der psychischen Belastung über die Zeit

**Figure 2:**
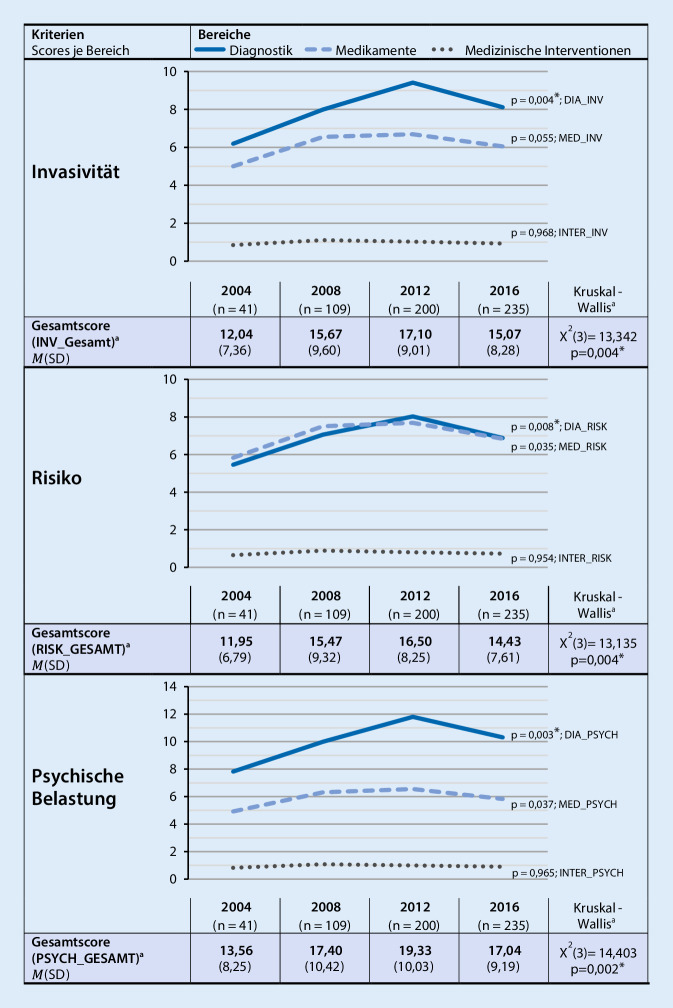

Im Hinblick auf die drei Kriterien **„*Invasivität“*, „*Risiko“*** und** „*psychische*** ***Belastung“*** von Maßnahmen, die vor Aufnahme zur multimodalen Schmerztherapie durchgeführt wurden, sind signifikante Veränderungen der Gesamtscores über die Zeit zu beobachten (Abb. 2). Betrachtet man die einzelnen Bereiche getrennt (nach „Diagnostik“, „Medikamente“ und „invasive Maßnahmen“) zeigt sich, dass diese Veränderungen durch den Bereich „**Diagnostik“** bedingt sind. Über alle Kriterien hinweg nehmen die Scores für „**Diagnostik“** von 2004 bis 2012 zu und anschließend leicht ab. Für die Bereiche **„Medikamente“** und **„medizinische Interventionen“ **lassen sich keine signifikanten Veränderungen feststellen (Abb. 2).

### Potenzielle Bedingungsfaktoren für Risikopatienten

Risikopatienten (Patienten, die „**hoch invasive**“ oder „**sehr risikoreiche**“ oder „**stark psychisch belastende**“ Maßnahmen erhalten hatten) weisen mehr schmerzbedingte Schulfehltage auf. Bei Patienten mit schmerzbedingten Schulfehltagen war die relative Wahrscheinlichkeit, dass „hoch invasive“ Maßnahmen zur Anwendung kamen, um 7 % erhöht. Gleiches gilt für die Anwendung „sehr risikoreicher“ (8 %) bzw. „stark psychisch belastender Maßnahmen“ (6 %). Maßgebend für diese Ergebnisse sind die Bereiche „Diagnostik“ und „Medikamente“ (Tab. 5). Insgesamt lagen 28 % aller Patienten in mindestens einem der Bereiche über dem Cut-off für Risikopatienten, 25 % in mindestens zwei und 22 % in allen drei Bereichen.

**Table 5:** 

Wert >75 %-Perzentil (ja) *N* = 585		Bedingungsfaktoren	Odds Ratio	95 %-KI	*p des *Modells	Cohen (*f*)
*Gesamt*^*a*^	Invasivität	Hauptschmerzort: Kopf	0,612	[0,386–0,971]	*X*^*2*^*(2)* *=* *18,974* *p* *<* *0,001*	0,27
		Schulfehltage (ja)	1,070	[1,034–1,106]		
	Risiko	Hauptschmerzort: Extremitäten	1,787	[1,040–3,072]	*X*^*2*^*(2)* *=* *21,592* *p* *<* *0,001*	0,29
		Schulfehltage (ja)	1,080	[1,044–1,118]		
	Psychische Belastung	Hauptschmerzort: Kopf	0,600	[0,378–0,952]	*X*^*2*^*(2)* *=* *16,679* *p* *<* *0,001*	0,25
		Schulfehltage (ja)	1,063	[1,028–1,100]		
*Diagnostik*	Invasivität	Hauptschmerzort: Bauch	3,484	[2,009–6,041]	*X*^*2*^*(4)* *=* *39,715* *p* *<* *0,001*	0,41
		Hauptschmerzort: Extremitäten	2,302	[1,290–4,109]		
		Schulfehltage (ja)	1,064	[1,027–1,103]		
		DIKJ T‑Wert ≥60 (ja)	0,533	[0,300–0,946]		
	Risiko	Hauptschmerzort: Kopf	0,371	[0,229–0,601]	*X*^*2*^*(2)* *=* *25,503* *p* *<* *0,001*	0,32
		Schulfehltage (ja)	1,058	[1,022–1,095]		
	Psychische Belastung	Hauptschmerzort: Kopf	0,476	[0,295–0,769]	*X*^*2*^*(3)* *=* *26,070* *p* *<* *0,001*	0,32
		Schulfehltage (ja)	1,063	[1,027–1,101]		
		DIKJ T‑Wert ≥60 (ja)	0,520	[0,294–0,922]		
*Medikamente*	Invasivität	Hauptschmerzort: Bauch	0,304	[0,156–0,591]	*X*^*2*^*(2)* *=* *32,381* *p* *<* *0,001*	0,36
		Schulfehltage (ja)	1,085	[1,041–1,123]		
	Risiko	Hauptschmerzort: Bauch	0,253	[0,123–0,523]	*X*^*2*^*(3)* *=* *32,406* *p* *<* *0,001*	0,36
		PPDI (Spanne 12–60)	1,034	[1,004–1,064]		
		Schulfehltage (ja)	1,056	[1,018–1,094]		
	Psychische Belastung	Hauptschmerzort: Bauch	0,282	[0,140–0,568]	*X*^*2*^*(2)* *=* *29,351* *p* *<* *0,001*	0,34
		Schulfehltage (ja)	1,078	[1,041–1,116]		
*Medizinische Interventionen*	Invasivität	DIKJ T‑Wert ≥60 (ja)	0,575	[0,339–0,976]	*X*^*2*^*(1)* *=* *4,451* *p* *=* *0,035*	0,13
	Risiko	DIKJ T‑Wert ≥60 (ja)	0,556	[0,328–0,942]	*X*^*2*^*(1)* *=* *5,059* *p* *=* *0,024*	0,14
	Psychische Belastung	Hauptschmerzort: Kopf	0,294	[0,146–0,592]	*X*^*2*^*(1)* *=* *13,027* *p* *<* *0,001*	0,26

^a^Scores der Bereiche Diagnostik, Medikamente und medizinische Interventionen zusammengefasst

Darüber hinaus kommt der Lokalisation des Hauptschmerzorts eine übergreifende Bedeutung zu. Kopfschmerzpatienten erhalten deutlich weniger invasive, gefährdende oder psychisch belastende Maßnahmen als Kinder mit andernorts lokalisierten Schmerzen. Bei Kindern mit Bauchschmerzen sowie Schmerzen in den Extremitäten steigt das relative Risiko invasiver diagnostischer Maßnahmen um das 3,5- bzw. 2,3-fache (Tab. 5).

Schmerzpatienten, bei denen eine klinisch relevante depressive Beeinträchtigung vorlag (DIKJ T‑Wert ≥60), hatten in der Vergangenheit mit einer relativen Wahrscheinlichkeit von 88 % eine weniger invasive wie psychisch gefährdende Diagnostik (92 %). Vergleichbares zeigt sich im Hinblick auf medizinische Interventionen: bei Vorliegen auffälliger Depressionswerte war die Anwendung invasiver bzw. risikoreicher Interventionen um 74 % bzw. 80 % weniger wahrscheinlich (Tab. 5). Für eine detaillierte Auflistung aller im Rahmen der logistischen Regression ausgeschlossenen Variablen siehe im elektronischen Zusatzmaterial, Tab. S4.

## Diskussion

Die vorliegende Studie belegt, dass die in den USA zu beobachtende Zunahme von invasiven, risikoreichen und psychisch belastenden diagnostischen und therapeutischen Interventionen bei chronisch funktionellen Schmerzen im Kindes- und Jugendalter in Deutschland nur von 2004 bis 2012, aber nicht zwischen 2012 und 2016 zu beobachten ist. Für diesen Verlauf sind vor allem die angewandten diagnostischen Maßnahmen verantwortlich. Es lassen sich Risikopatienten identifizieren, die bei einer funktionellen Störung „hoch invasive“, „sehr risikoreiche“ oder „stark psychisch belastende“ Diagnostik erhalten. Dies sind Kinder mit hoher schmerzbedingter Beeinträchtigung (viele Schulfehltage), die an Bauch oder Muskel- und Gelenkschmerzen leiden. Das Vorliegen von Kopfschmerzen und einer Depressivität scheinen vor (zu) viel Diagnostik und (zu) invasiver Schmerztherapie zu schützen.

### Warum erfolgt bei funktionellen Schmerzen im Kindes- und Jugendalter eine so umfangreiche Diagnostik und welche Faktoren sind dafür verantwortlich?

Bei den meisten pädiatrischen Patienten ist „Schmerz“ ein Symptom und keine eigenständige Krankheit. In der Primärversorgung gilt es, das Symptom Schmerz ernst zu nehmen, Notfälle zu erkennen und verantwortliche Grunderkrankungen auszuschließen. In der Regel sind hierfür eine sorgfältige Anamnese und eine gründliche körperliche Untersuchung ausreichend [9]. Lässt sich die Ursache der Schmerzen nicht detektieren, sind hierfür zwei Erklärungsmodelle denkbar. Erstens: Den Schmerzen liegt eine einzige diagnostizierbare Ursache zugrunde. Die bisher stattgefundene Diagnostik war nicht umfangreich oder gezielt genug, um diese zu detektieren (monokausaler Ansatz). Zweitens: Der persistierende oder rezidivierende Schmerz liegt ohne einen zugrunde liegenden Organschaden vor und muss biopsychosozial betrachtet werden (multikausaler Ansatz).

### Folgen des monokausalen Ansatzes

In Folge des ersten Erklärungsmodells einer einzigen vermuteten Ursache scheinen sich Ärztinnen und Ärzte manchmal einer Vielzahl diagnostischer Möglichkeiten zu bedienen, unabhängig gültiger Leitlinien und der Tragweite der jeweiligen Diagnostik für den Patienten. Am ehesten geschieht dies in der Pädiatrie aus der Sorge, gravierende und potenziell kurativ behandelbare Ursachen zu übersehen. Auch Patienten oder deren Eltern scheint ein monokausales Erklärungsmodell chronischer Schmerzen oft plausibel; meist aus Angst und Verunsicherung, da auch sie Schmerz in der Regel als Symptom einer zugrunde liegenden Erkrankung kennen und nicht als eigenständige Erkrankung [25, 26]. Häufig werden dann mehrere Ärzte aufgesucht, in der Hoffnung, „die eine“ körperliche Ursache zu finden [16, 27, 28]. Eine derart ausgedehnte Diagnostik kann zur Verstärkung des Schmerzproblems im Sinne einer iatrogenen Chronifizierung beitragen [9, 10, 29]. Die Vorstellung (nur) einer organisch begründbaren Ursache für die Schmerzen wird oft durch die Haltung verstärkt, ausschließlich biologisch orientierte Schmerztherapien seien wirksam, selbst wenn die genaue Schmerzursache noch nicht feststeht. In dessen Folge werden von Behandlern, Eltern oder pädiatrischen Patienten selber medikamentöse oder invasive Therapie „probatorisch“ durchgeführt (z. B. protrahierte Selbstmedikation). Diesen Therapien wohnen zahlreich potenzielle Nebenwirkungen und Komplikationen inne [30]. Mindestens 20 % medizinischer Maßnahmen bei der Behandlung chronischer Schmerzen sind überflüssig [31]. Die in vielen Fällen bestehende geringe Evidenz zur Wirksamkeit von (neuen) Maßnahmen v. a. im Bereich Pharmakotherapie scheint Behandler vor der Anwendung dieser Therapien bei chronischen Schmerzen nicht generell abzuschrecken [16, 32]. Letztlich mitbestimmend im Entscheidungsprozess sind die persönlichen Wertvorstellungen der Behandelnden [33].

Als möglicher Bedingungsfaktor für diagnostische wie medikamentöse Maßnahmen konnte neben der Anzahl an Schulfehltagen der Hauptschmerzort identifiziert werden. Letzteres lässt sich möglicherweise darauf zurückführen, dass sich die mögliche Anzahl diagnostischer Maßnahmen zur Differenzialdiagnose von Bauchschmerzen und Kopfschmerzen stark unterscheidet. Die Mehrzahl somatischer Ursachen für Kopfschmerzen wird durch die Anwendung einer Magnetresonanztomographie (MRT) ausgeschlossen; bei chronischen Bauchschmerzen kann eine Vielzahl (invasiver) diagnostischer Maßnahmen von der Sonographie, über das MRT bis zur Laparoskopie durchgeführt werden. Geht man davon aus, dass das Ausmaß der schmerzbedingten Beeinträchtigung den Druck auf die Ärztin/den Arzt erhöht, jetzt „endlich etwas zu tun“, erklärt dies, warum viele Schulfehltage viel Diagnostik und entsprechend auch eine höhere Gefährdung nach sich ziehen. Gleichzeitig könnten die Ergebnisse darauf hinweisen, dass durch viel Diagnostik und Therapie und eine entsprechend hohe Invasivität der Maßnahmen, die Funktionalität der Patienten in Form reduzierter Schulfähigkeit negativ beeinträchtigt wird. Aufgrund des Querschnittsdesigns der vorliegenden Studie kann die Richtung dieses Zusammenhangs nicht abschließend geklärt werden.

### Die multikausale Betrachtung chronischer Schmerzen

Das zweite Erklärungsmodell, dass persistierender oder rezidivierender Schmerz auch ohne einen zugrunde liegenden Organschaden auftreten kann und biopsychosozial betrachtet werden muss, wird von vielen aktuellen Leitlinien unterstützt [34, 35]. Der ab 2012 einsetzende abnehmende Trend aller im Vorfeld einer spezialisierten stationären Schmerztherapie angewandten Maßnahmen könnte durch die Verbreitung von Leitlinien sowie einen wachsenden Zuspruch zur evidenzbasierten Medizin (EBM) im Sinne eines Choosing-wisely-Effekts erklärt werden. Beispielsweise wird die MRT gegenüber der Computertomographie (CT) zunehmend als bevorzugtes Mittel bildgebender Kopfschmerzdiagnostik gewählt [36]. Positiv ist ebenfalls, dass sich – anders als in den USA – eine Abnahme der Verordnung von Opioiden bei den pädiatrischen Patienten mit funktionellen Schmerzen zeigt. Insbesondere vor dem Hintergrund, dass vor allem bei unbegründeter Indikation die Einnahme von Opioiden während der Adoleszenz mit einem erhöhten Risiko des Substanzmissbrauchs im Erwachsenenalter als auch schwerwiegender Beeinträchtigung des sozialen Lebens wie zum Beispiel längerer Arbeitslosigkeit assoziiert ist [37–40].

### Einschränkungen der Studie

Letztlich sind die vorliegenden Ergebnisse vor dem Hintergrund der potenziell bestehenden Unvollständigkeit von Patientenakten zu betrachten. Dies kann unter anderem auf eine unpräzise Rekonstruktion der bereits erhaltenen Maßnahmen durch Eltern und Patienten zurückgeführt werden. Die konkreten Fachbezeichnungen der verschiedenen Maßnahmen sind für medizinische Laien nicht immer gegenwärtig und die vorausgegangenen mitunter „langen Schmerzkarrieren“ erschweren, sich an die korrekte Anzahl an erhaltenen Maßnahmen zu erinnern [16]. Auch die variierende Anzahl betrachteter Maßnahmen (*n* Diagnostik = 21, Medikamente = 17, medizinische Interventionen = 13) sowie die Spezifität insbesondere der medizinischen Interventionen sind als Limitationen zu werten. Die Bewertung der Indikation von Maßnahmen wie auch ihrer Häufigkeit ohne eindeutige Kausalkette, die zu deren Anwendung geführt hat, ist im Nachgang nicht leistbar. Die Einordnung der Maßnahmen hinsichtlich der drei Kriterien (Invasivität, Risiko, psychische Belastung) sollte unter Berücksichtigung weiterer Experten revalidiert werden. Auch muss geprüft werden, ob die Betrachtung der Einnahmedauer eines Medikaments einen Einfluss auf die Ergebnisse hat. Ein prospektiver Ansatz wäre hier zielführender. Eine weitere Limitation der vorliegenden Studie stellt die vergleichsweise geringe Fallzahl im Jahr 2004 dar. Darüber hinaus basiert die vorliegende Studie nur auf Daten einer einzigen spezialisierten tertiären Versorgungseinrichtung, was die Generalisierbarkeit einschränkt. Zukünftige Studien sollten daher Daten aus mehreren tertiären Versorgungseinrichtungen einschließen, die auf die Behandlung chronischer Schmerzen bei Kindern und Jugendlichen spezialisiert sind und auch Routinedaten der Krankenkassen berücksichtigen.

## Resümee

Einige Patientinnen und Patienten haben ein höheres Risiko für potenziell gesundheitsgefährdende Maßnahmen. Generell zeigt sich jedoch zwischen 2012 und 2016 der Trend einer abnehmenden Prävalenz der Anzahl angewandter Maßnahmen – insbesondere der Diagnostik. Es bleibt zu beobachten, ob sich dieser Trend auch nach 2016 weiter fortsetzt. Zur weiterführenden Klärung der Frage, welche weiteren Faktoren und deren Konstellation auf Patientenseite (Bildungsniveau, Erwartung von Eltern und Patient) bzw. aufseiten der Ärztinnen und Ärzte (Lebensalter, Geschlecht, Zusatzweiterbildungen) den Behandlungsprozess mit beeinflussen, könnten experimentelle Studien, in denen Patientenfälle systematisch variiert werden, herangezogen werden.

## Fazit für die Praxis

- Anzahl und Ausmaß diagnostischer und therapeutischer Maßnahmen nahmen bei Kindern mit chronischen funktionellen Schmerzen in den Jahren 2004 bis 2012 zu.
- Es wurden zunehmend invasivere, risikoreichere und die Psyche stark belastende Maßnahmen durchgeführt; dieser Trend ist zwischen 2012 und 2016 wieder rückläufig.
- Im internationalen Vergleich z. B. zur USA wird eine positive Entwicklung bei der Verordnung von Medikamenten – insbesondere eine Abnahme der Opioidverschreibungen für chronisch funktionelle Schmerzen beobachtet.
- Kinder mit Bauch- bzw. Gliederschmerzen und solche, mit hoher schmerzbedingter Beeinträchtigung (z. B. viele Schulfehltage), sind besonders gefährdet für hoch invasive diagnostische Maßnahmen.
- Eine leitliniengerechte Behandlung chronisch funktioneller Beschwerden im Kindesalter setzt die Betrachtung dieser im Sinne des biopsychosozialen Modells voraus.

## Caption Electronic Supplementary Material








