# Supplementary material for: Diagnostics and therapy in children and adolescents with chronic pain: Trends in interventions potentially dangerous to health
Source: Schmerz. 2020 Nov 13;35(2):83–93. [Article in German] doi: 10.1007/s00482-020-00506-5 (PMC7997831; doi:10.1007/s00482-020-00506-5)
Supplement: Supplementary file 1 [file 482_2020_506_MOESM1_ESM.pdf]

Tab. S1 Bewertung der Invasivität, des Risikos und der psychische Belastung ausgewählter Maßnahmen durch Experten[1] (anhand 5-stufiger Likert-Skala; 1-5)[2]

**Art der Maßnahme**	**Maßnahme bzw. Wirkstoff(-Klasse)** (alphabetisch)	**Invasivität** [3] (*M*)	**Risiko** [4] (*M*)	**Psychische Belastung** [5] (*M*)
**Diagnostik** (n = 21)	Computertomographie	2,39	2,62	2,69
	Echokardiographie	1,33	1,08	1,83
	Elektroenzephalogramm (EEG)	1,50	1,25	2,17
	Elektrokardiogramm (EKG)	1,42	1,25	1,83
	Elektrophysiologische Untersuchung (EPU)	2,00	1,55	2,60
	Koloskopie	3,75	2,83	3,92
	Laparoskopie	4,67	3,33	4,42
	Lumbalpunktion	4,08	3,17	4,33
	Lungenfunktionstest	1,64	1,18	2,09
	Miktionszystourethrogramm (MCU)	3,42	2,83	3,75
	Magnetresonanztomographie (MRT)	2,23	1,85	3,23
	Magnetresonanztomographie (MRT) mit Kontrastmittel	2,83	2,50	3,17
	Ösophago-Gastro-Duodenoskopie (ÖGD)	3,75	2,75	3,83
	Positronen-Emissions-Tomographie (PET)	2,82	2,55	3,00
	Quantitative Sensorische Testung (QST)	1,70	1,40	1,89
	Röntgen	2,08	2,39	2,08
	Sonographie	1,31	1,08	1,62
	Szintigrafie	2,82	2,91	2,91
	Wasserstoffatemtests	1,70	1,50	2,10
	Zystoskopie	3,73	3,10	4,00
	Zystomannometrie	3,33	2,89	3,78
**Medikamente** (n = 17)	Antidepressiva	2,90	3,33	2,80
	Antiemetika	2,20	2,56	2,20
	Acetylsalicylsäure (ASS)	2,08	3,00	2,09
	Benzodiazepine	3,00	3,50	2,90
	Gabapentin	2,43	2,71	2,57
	Hochpotente Opioide [6]	3,09	3,36	2,82
	Homöopathika	1,67	1,75	1,92
	Ibuprofen	2,08	2,33	2,00
	Metamizol	2,33	2,58	2,09
	Niederpotente Opioide [7]	2,64	2,91	2,55
	Paracetamol	2,00	2,58	2,00
	Protonenpumpenhemmer	2,30	2,33	2,30
	Pregabalin	2,71	2,71	2,57
	Spasmolytika	2,55	2,55	2,27
	Steroide	3,10	3,20	3,10
	Topiramat	2,50	2,75	2,50
	Triptane	2,46	2,27	2,27

**Medizinische Interventionen** (n = 13)	Akupunktur	2,33	1,75	2,42
	Appendektomie	4,25	3,33	3,50
	Arthroskopie	4,25	3,33	3,75
	Biopsie	3,67	2,50	3,58
	Cerebralshuntanlage	4,82	4,18	4,46
	Infiltrationsanästhesie	3,27	2,64	3,27
	Intraartikuläre Injektionen	3,82	3,00	3,55
	Nervenblockade	3,60	3,30	3,30
	Oberflächenanästhesie	2,17	2,08	2,17
	Peri-/Epiduralanästhesie	4,00	3,20	3,82
	Plexusblockade	4,00	3,50	3,60
	Schmerzpumpe	3,50	2,70	2,90
	Spinalanästhesie	4,00	3,27	3,64

[1] N = 13, bestehend aus ärztlichen (Anästhesie/Schmerzmedizin, Kinderrheumatologie/-Immunologie, Neuropädiatrie, Kinder-/Jugendpsychiatrie, Kinderchirurgie/-Orthopädie, Kinderradiologie) und nicht-ärztlichen (Patienten/-Angehörige, Pflegewissenschaften, Physiotherapie, MFA) Experten; [2] Dimension bzw. Ausprägung des jeweiligen Items: 1 (kein), 2 (gering), 3 (mittel), 4 (hoch), 5 (sehr hoch), zzgl. 0 (keine Einschätzung möglich) ; [3] **Invasivität** wird hier definiert als Umfang der Integritätsverletzung des Körpers, [4] **Risiko** als die Wahrscheinlichkeit für das Auftreten und die Ausprägung unerwünschter Nebeneffekte und [5] **psychische Belastung** als Grad der durch die Maßnahme bedingten Beeinträchtigung der Psyche; [6] Analgetische Potenz ≥ 1; [7] Analgetische Potenz < 1
