# Supplementary material for: Diagnostics and therapy in children and adolescents with chronic pain: Trends in interventions potentially dangerous to health
Source: Schmerz. 2020 Nov 13;35(2):83–93. [Article in German] doi: 10.1007/s00482-020-00506-5 (PMC7997831; doi:10.1007/s00482-020-00506-5)
Supplement: Supplementary file 2 [file 482_2020_506_MOESM2_ESM.pdf]

Tab. S2 Übersicht angewendeter Maßnahmen der Diagnostik nach Jahren

**Diagnostische Maßnahme** N[1] (%)[2]	**2004** (*n* = 41)	**2008** (*n* = 109)	**2012** (*n* = 200)	**2016** (*n* = 235)	∑ (*N* = 585)	Chi[2]
Computertomographie	6 (14,6)	18 (16,8)	13 (6,5)	14 (6,0)	51 (8,7)	$\chi^2(3)=14{,}077$ $p=0{,}003$
Echokardiographie	2 (4,9)	7 (6,5)	16 (8,0)	13 (5,5)	38 (6,5)	$\chi^2(3)=1{,}279$ $p=0{,}734$
Elektroenzephalogramm (EEG)	19 (46,3)	52 (48,6)	92 (46,0)	96 (40,9)	259 (44,4)	$\chi^2(3)=2{,}232$ $p=0{,}526$
Elektrokardiogramm (EKG)	6 (14,6)	13 (12,0)	27 (13,5)	29 (12,3)	75 (12,8)	$\chi^2(3)=0{,}310$ $p=0{,}958$
Elektrophysiologische Untersuchung (EPU)	2 (4,9)	10 (9,3)	20 (10,0)	18 (7,7)	50 (8,6)	$\chi^2(3)=1{,}551$ $p=0{,}671$
Koloskopie	2 (4,9)	5 (4,7)	23 (11,5)	19 (8,1)	49 (8,4)	$\chi^2(3)=5{,}118$ $p=0{,}163$
Laparoskopie	0 (0)	2 (1,8)	5 (2,5)	5 (2,1)	12 (2,1)	$\chi^2(3)=1{,}091$ $p=0{,}779$
Lumbalpunktion	4 (9,8)	12 (11,1)	13 (6,5)	22 (9,4)	51 (8,7)	$\chi^2(3)=2{,}188$ $p=0{,}534$
Lungenfunktionstest	0 (0)	1 (0,9)	2 (1,0)	1 (0,4)	4 (0,7)	$\chi^2(3)=0{,}895$ $p=0{,}827$
Miktionszystourethrogramm (MCU)	0 (0)	1 (0,9)	1 (0,5)	1 (0,4)	3 (0,5)	$\chi^2(3)=0{,}597$ $p=0{,}897$
Magnetresonanztomographie (MRT)	23 (56,1)	79 (73,8)	163 (81,5)	200 (85,1)	465 (79,8)	$\chi^2(3)=21{,}086$ $p<0{,}001$
Magnetresonanztomographie (MRT) mit Kontrastmittel	0 (0)	7 (6,5)	23 (11,6)	24 (10,2)	54 (9,3)	$\chi^2(3)=6{,}617$ $p=0{,}085$
Ösophago-Gastro-Duodenoskopie (ÖGD)	3 (7,3)	12 (11,2)	39 (19,6)	39 (16,6)	96 (16,0)	$\chi^2(3)=6{,}108$ $p=0{,}106$
Positronen-Emissions-Tomographie (PET)	0 (0)	1 (0,9)	0 (0)	0 (0)	1 (0,2)	$\chi^2(3)=4{,}374$ $p=0{,}224$
Quantitative Sensorische Testung (QST)	0 (0)	0 (0)	1 (0,5)	0 (0)	1 (0,2)	$\chi^2(3)=1{,}928$ $p=0{,}587$
Röntgen	11 (26,8)	39 (36,4)	53 (26,5)	54 (23,0)	157 (26,9)	$\chi^2(3)=6{,}810$ $p=0{,}078$
Sonographie	10 (24,4)	34 (32,1)	76 (38,2)	71 (30,2)	191 (32,9)	$\chi^2(3)=4{,}671$ $p=0{,}197$
Szintigrafie	2 (4,9)	4 (3,7)	14 (7,0)	2 (0,9)	22 (3,8)	$\chi^2(3)=11{,}439$ $p=0{,}010$
Wasserstoffatemtests	2 (4,9)	6 (5,6)	31 (15,5)	21 (8,9)	60 (10,3)	$\chi^2(3)=10{,}285$ $p=0{,}016$
Zystoskopie	0 (0)	1 (0,9)	0 (0)	1 (0,4)	2 (0,3)	$\chi^2(3)=1{,}935$ $p=0{,}586$
Zystomannometrie	0 (0)	0 (0)	0 (0)	1 (0,4)	1 (0,2)	$\chi^2(3)=1{,}492$ $p=0{,}684$

[1] Anzahl Patienten, die Maßnahme erhalten haben (binär-nomiales Messniveau); [2] gültige Prozent
