# Supplementary material for: Diagnostics and therapy in children and adolescents with chronic pain: Trends in interventions potentially dangerous to health
Source: Schmerz. 2020 Nov 13;35(2):83–93. [Article in German] doi: 10.1007/s00482-020-00506-5 (PMC7997831; doi:10.1007/s00482-020-00506-5)
Supplement: Supplementary file 3 [file 482_2020_506_MOESM3_ESM.pdf]

Tab. S3 Übersicht angewendeter medizinischer Interventionen nach Jahren

**Medizinische Intervention** N[1] (%)[2]	**2004** (*n* = 41)	**2008** (*n* = 109)	**2012** (*n* = 200)	**2016** (*n* = 235)	∑ (*N* = 585)	Chi²
Akupunktur	7 (17,1)	18 (16,5)	34 (17,0)	37 (15,7)	96 (16,4)	$\chi^2(3)=0,141$ $p=0,987$
Appendektomie	2 (4,9)	3 (2,8)	8 (4,0)	6 (2,6)	19 (3,2)	$\chi^2(3)=1,153$ $p=0,764$
Arthroskopie	0 (0)	2 (1,8)	1 (0,5)	2 (0,9)	5 (0,9)	$\chi^2(3)=1,886$ $p=0,596$
Biopsie	0 (0)	0 (0)	5 (2,5)	4 (1,7)	9 (1,5)	$\chi^2(3)=3,606$ $p=0,307$
Cerebralshunt	0 (0)	0 (0)	1 (0,5)	0 (0)	1 (0,2)	$\chi^2(3)=1,928$ $p=0,587$
Infiltrationsanästhesie	1 (2,4)	6 (5,5)	5 (2,5)	12 (5,1)	24 (4,1)	$\chi^2(3)=2,740$ $p=0,433$
Intraartikuläre Injektionen	0 (0)	0 (0)	2 (1,0)	4 (1,7)	6 (1,0)	$\chi^2(3)=2,615$ $p=0,455$
Nervenblockade	0 (0)	2 (1,8)	4 (2,0)	4 (1,7)	10 (1,7)	$\chi^2(3)=0,824$ $p=0,844$
Oberflächenanästhesie	0 (0)	3 (2,8)	3 (1,5)	3 (1,3)	9 (1,5)	$\chi^2(3)=1,809$ $p=0,613$
Peri-/Epiduralanästhesie	0 (0)	0 (0)	1 (0,5)	0 (0)	1 (0,2)	$\chi^2(3)=1,928$ $p=0,587$
Plexusblockade	0 (0)	2 (1,8)	0 (0)	0 (0)	2 (0,3)	$\chi^2(3)=8,764$ $p=0,033$
Schmerzpumpe	0 (0)	1 (0,9)	1 (0,5)	0 (0)	2 (0,3)	$\chi^2(3)=2,153$ $p=0,541$
Spinalanästhesie	0 (0)	1 (0,9)	0 (0)	0 (0)	1 (0,2)	$\chi^2(3)=4,374$ $p=0,224$

[1] Anzahl Patienten, die Maßnahme erhalten haben (binär-nominales Messniveau); [2] gültige Prozent
