# Supplementary material for: Diagnostics and therapy in children and adolescents with chronic pain: Trends in interventions potentially dangerous to health
Source: Schmerz. 2020 Nov 13;35(2):83–93. [Article in German] doi: 10.1007/s00482-020-00506-5 (PMC7997831; doi:10.1007/s00482-020-00506-5)
Supplement: Supplementary file 4 [file 482_2020_506_MOESM4_ESM.pdf]

Tab. S4 Übersicht im Rahmen logistischer Regression **ausgeschlossener** Variablen

**Score** (Cutoff >75%-Perzentil) N = 585		**Unabhängige Variable** (Prädiktoren)	***p***
**Diagnostik**	Invasivität	Alter bei Schmerzbeginn	0,948
		Geschlecht	0,083
		Versicherungsstatus	0,390
		IQ	0,679
		Schmerzdauer (in Monaten)	0,635
		Hauptschmerzort Kopf	0,159
		Hauptschmerzort Nacken	0,794
		Hauptschmerzort Rücken	0,490
		Hauptschmerzort Thorax	0,389
		PPDI (der letzten 4 Wochen)	0,725
		AFS mind. 1 Subtest über Cutoff	0,960
	Risiko	Alter bei Schmerzbeginn	0,530
		Geschlecht	0,858
		Versicherungsstatus	0,543
		IQ	0,908
		Schmerzdauer (in Monaten)	0,603
		Hauptschmerzort Nacken	0,245
		Hauptschmerzort Bauch	0,251
		Hauptschmerzort Rücken	0,201
		Hauptschmerzort Extremitäten	0,274
		Hauptschmerzort Thorax	0,225
		PPDI (der letzten 4 Wochen)	0,836
		DIKJ T-Wert $\geq$ 60	0,157
		AFS mind. 1 Subtest über Cutoff	0,971
	Psychische Belastung	Alter bei Schmerzbeginn	0,205
		Geschlecht	0,124
		Versicherungsstatus	0,518
		IQ	0,754
		Schmerzdauer (in Monaten)	0,217
		Hauptschmerzort Nacken	0,638
		Hauptschmerzort Bauch	0,226
		Hauptschmerzort Rücken	0,072
		Hauptschmerzort Extremitäten	0,950
		Hauptschmerzort Thorax	0,238
		PPDI (der letzten 4 Wochen)	0,453
		DIKJ T-Wert $\geq$ 60	0,024
		AFS mind. 1 Subtest über Cutoff	0,771
**Medikamente**	Invasivität	Alter bei Schmerzbeginn	0,967
		Geschlecht	0,254
		Versicherungsstatus	0,842
		IQ	0,389
		Schmerzdauer (in Monaten)	0,116
		Hauptschmerzort Kopf	0,892
		Hauptschmerzort Nacken	0,756
		Hauptschmerzort Rücken	0,995
		Hauptschmerzort Extremitäten	0,515
		Hauptschmerzort Thorax	0,814
		PPDI (der letzten 4 Wochen)	0,062
		DIKJ T-Wert $\geq$ 60	0,136
		AFS mind. 1 Subtest über Cutoff	0,256
	Risiko	Alter bei Schmerzbeginn	0,274
		Geschlecht	0,263
		Versicherungsstatus	0,468
		IQ	0,161
		Schmerzdauer (in Monaten)	0,302

		Hauptschmerzort Kopf	0,608
		Hauptschmerzort Nacken	0,453
		Hauptschmerzort Bauch	0,223
		Hauptschmerzort Rücken	0,378
		Hauptschmerzort Extremitäten	0,802
		Hauptschmerzort Thorax	0,411
		AFS mind. 1 Subtest über Cutoff	0,297
	Psychische Belastung	Alter bei Schmerzbeginn	0,644
		Geschlecht	0,303
		Versicherungsstatus	0,975
		IQ	0,460
		Schmerzdauer (in Monaten)	0,162
		Hauptschmerzort Kopf	0,290
		Hauptschmerzort Nacken	0,247
		Hauptschmerzort Rücken	0,299
		Hauptschmerzort Extremitäten	0,160
		Hauptschmerzort Thorax	0,868
		PPDI (der letzten 4 Wochen)	0,057
		DIKJ T-Wert ≥ 60	0,567
		AFS mind. 1 Subtest über Cutoff	0,242
**Medizinische Interventionen**	Invasivität	Alter bei Schmerzbeginn	0,274
		Geschlecht	0,446
		Versicherungsstatus	0,424
		IQ	0,224
		Schmerzdauer (in Monaten)	0,215
		Hauptschmerzort Kopf	0,785
		Hauptschmerzort Nacken	0,476
		Hauptschmerzort Bauch	0,270
		Hauptschmerzort Rücken	0,326
		Hauptschmerzort Extremitäten	0,702
		Hauptschmerzort Thorax	0,396
		PPDI (der letzten 4 Wochen)	0,078
		Schmerzbezogene Schulfehltage (der letzten 4 Wochen)	0,264
		AFS mind. 1 Subtest über Cutoff	0,300
	Risiko	Alter bei Schmerzbeginn	0,227
		Geschlecht	0,319
		Versicherungsstatus	0,347
		IQ	0,207
		Schmerzdauer (in Monaten)	0,303
		Hauptschmerzort Kopf	0,722
		Hauptschmerzort Nacken	0,841
		Hauptschmerzort Bauch	0,148
		Hauptschmerzort Extremitäten	0,090
		Hauptschmerzort Thorax	0,582
		Schmerzbezogene Schulfehltage (der letzten 4 Wochen)	0,270
		PPDI (der letzten 4 Wochen)	0,865
		DIKJ T-Wert ≥ 60	0,616
		AFS mind. 1 Subtest über Cutoff	0,344
	Psychische Belastung	Alter bei Schmerzbeginn	0,820
		Geschlecht	0,900
		Versicherungsstatus	0,194
		IQ	0,496
		Schmerzdauer (in Monaten)	0,063
		Hauptschmerzort Nacken	0,262
		Hauptschmerzort Bauch	0,417
		Hauptschmerzort Rücken	0,275
		Hauptschmerzort Thorax	0,567

		Schmerzbezogene Schulfehltage (der letzten 4 Wochen)	0,278
		PPDI (der letzten 4 Wochen)	0,254
		DIKJ T-Wert ≥ 60	0,160
		AFS mind. 1 Subtest über Cutoff	0,786
**Gesamt** (Maßnahmen insgesamt)	Invasivität	Alter bei Schmerzbeginn	0,652
		Geschlecht	0,876
		Versicherungsstatus	0,953
		IQ	0,689
		Schmerzdauer (in Monaten)	0,232
		Hauptschmerzort Nacken	0,498
		Hauptschmerzort Bauch	0,600
		Hauptschmerzort Rücken	0,631
		Hauptschmerzort Extremitäten	0,505
		Hauptschmerzort Thorax	0,907
		PPDI (der letzten 4 Wochen)	0,229
		DIKJ T-Wert ≥ 60	0,269
		AFS mind. 1 Subtest über Cutoff	0,537
	Risiko	Alter bei Schmerzbeginn	0,520
		Geschlecht	0,650
		Versicherungsstatus	0,883
		IQ	0,563
		Schmerzdauer (in Monaten)	0,311
		Hauptschmerzort Kopf	0,375
		Hauptschmerzort Nacken	0,959
		Hauptschmerzort Bauch	0,175
		Hauptschmerzort Rücken	0,890
		Hauptschmerzort Thorax	0,891
		PPDI (der letzten 4 Wochen)	0,282
		DIKJ T-Wert ≥ 60	0,495
		AFS mind. 1 Subtest über Cutoff	0,474
	Psychische Belastung	Alter bei Schmerzbeginn	0,960
		Geschlecht	0,580
		Versicherungsstatus	0,674
		IQ	0,549
		Schmerzdauer (in Monaten)	0,345
		Hauptschmerzort Nacken	0,969
		Hauptschmerzort Bauch	0,547
		Hauptschmerzort Rücken	0,898
		Hauptschmerzort Extremitäten	0,528
		Hauptschmerzort Thorax	0,896
		PPDI (der letzten 4 Wochen)	0,483
		DIKJ T-Wert ≥ 60	0,308
		AFS mind. 1 Subtest über Cutoff	0,640
